# Supplementary material for: Synergistic Efficacy of Policosanol (Raydel®) and Banaba Leaf Extract to Treat Hyperglycemia and Dyslipidemia in Streptozotocin-Induced Diabetic and Hyperlipidemic Zebrafish (Danio rerio): Protection of Liver and Kidney with Enhanced Tissue Regeneration
Source: Pharmaceuticals (Basel). 2025 Mar 3;18(3):362. doi: 10.3390/ph18030362 (PMC11946653; doi:10.3390/ph18030362)
Supplement: Supplementary file 1 [file pharmaceuticals-18-00362-s001.zip › pharmaceuticals-3474098-supplementary.pdf]

Supplementary Material

GSTIN. 08AAACU7574J1ZU

Umalaxmi Organics Pvt. Ltd.

A Government Recognised Export House

"An ISO 9001:2008 GMP, HACCP, STAR K KOSHER, HALAL & Organic Certified Company"

Factory : F-245-249 & 255, Agro Food Park, Boranada, Jodhpur - 342 012, Rajasthan, INDIA

Tel. No. : +91 - 2931-281204, 281202,281207 Fax No. : +91 - 2931 - 281201

E-mail : info@umalaxmi-organics.com Website : www.umalaxmi-organics.com

CIN. No. U01122RJ2005PTC020926

Certificate of Analysis

Month of Mfg.: FEBRUARY, 2024
Month of Exp.: JANUARY, 2027

Products Name:	Banaba Dry Extract
Latin Name:	***Lagerstroemia speciosa***
Raw Material Group:	Leaves
Batch No:	**UO/LSD-1825/02/23-24**
Quantity:	500KG (FROM 1000 KG BATCH)
Country of Origin:	Made in India

ITEMS	SPECIFICATIONS	TEST RESULT	TEST METHOD
Appearance:	Fine Powder	Complies	Visual
Color:	Light Brown to Dark Brown Color Powder	Complies	Visual
Odor:	Characteristic	Complies	Organoleptic
Taste:	Characteristic	Complies	Organoleptic

ITEMS	SPECIFICATIONS	TEST RESULT	TEST METHOD
Method of Extraction:	Hydro-Alcoholic	Complies	In-House
Mesh Size:	NLT 90% through 40 mesh	99.5%	Sieve screen
Loss on Drying:	NMT 8.0%	2.59%	USP/Karl Fischer
Bulk Density: Tapped	0.20- 1.2g/ml	0.744	USP/AOAC
Assay – Corosolic Acid:	NLT 1.0% w/w	1.15%	By HPLC
Heavy Metals:	NMT 10 ppm	Complies	ICP/MS
Arsenic:	NMT 2 ppm	0.012 ppm	ICP/MS
Cadmium:	NMT 0.5 ppm	BLQ	ICP/MS
Lead:	NMT 2 ppm	0.026 ppm	ICP/MS
Mercury:	NMT 0.2 ppm	BLQ	ICP/MS

MICROBIOLOGY

Total Plate Count:	NMT 10,000cfu/g	<10cfu/g	AOAC, BAM
Total Yeast & Mold:	NMT 1,000cfu/g	<10cfu/g	AOAC, BAM
E. Coli:	Absent	Absent	AOAC, BAM
Salmonella:	Absent	Absent	AOAC, BAM
Staphylococcus aureus:	Negative in 25 g	Negative	AOAC, BAM
Genetic Modification	GMO FREE	Complies	
BSE/TSE STATUS	To Comply with USP	Complies	
Sterilization:	This product has been treated by heat/steam only		
Storage:	Store in a well-closed container away from moisture, sunlight, and heat.		
Shelf Life:	Re-test 3 years from the date of manufacture.		
Kosher Certi.:	This product is KOSHER certified & Kosher certificate available on request.		
Allergen Statement	Enclosed		

The product submitted complies with the prescribed standards.

ANALYSED BY

APPROVED BY

UMALAXMI ORGANICS PVT. LTD.

U. L. O.

Registered Office : Dave Bhawan, Mod Bhatta, Industrial Area, Sojat City, Pali - 306104 (Raj.)
Branch Office : 701-702, 7th Floor, Sidharth Complex, R.C. Dutt Rd. Alkapuri
Vadodara - 390 007. Gujarat, India.
Telephone : 0091 - 265 - 2351536, 2326019 Fax : 0091 - 265 - 2336684
Email : documents@umalaxmi-organics.com accounts@umalaxmi-organics.com

Supplementary Table S1: A certificate of analysis of the used banaba extract.

Supplementary Method S1:

Quantification of blood lipoprotein profile hepatic function biomarkers AST and ALT

Blood (2 μL) was drawn from the hearts of the adult fish, combined with 3 μL of phosphate-buffered saline (PBS)-ethylenediaminetetraacetic acid (EDTA, final concentration, 1 mM) and then collected in EDTA-treated tubes. The plasma total cholesterol (TC) and triglycerides (TGs) were determined using commercial assay kits (cholesterol, T-CHO, and TGs, Cleantech TS-S; Wako Pure Chemical, Osaka, Japan) as per the method suggested by the suppliers. In brief, 5 μL serum was mixed with 200 μL reaction mixture (supplied with a commercial assay kit) for the TC analysis. The content was incubated at 37°C for 10 min, resulting in a red-colored product quantified by adsorption at 490 nm (Microplate reader, Bio-Rad, Hercules, CA, USA).

Similarly, 5 μL serum was mixed with a 200 μL of TGs-specific reaction mixture (supplied with a commercial assay kit) for TGs analysis. The content was incubated for 10 min at 37°C, and the formed colored product was quantified by taking adsorption at 490 nm.

For HDL-C analysis, serum was mixed in an equal ratio with the separation solution (supplied with a commercial assay kit), followed by centrifugation at 3,000 rpm for 10 min. The supernatant (20 μL) was collected and blended with a 200 μL reaction mixture (supplied with a commercial assay kit). After 10 min incubation at 37°C, red color intensity corresponding to HDL-C was quantified by taking absorption at 490 nm.

The LDL-C level was quantified using the Friedewald equation:

$$\text{LDL-C}=\text{TC}-\text{HDL-C}-(\text{TG}/5)$$

The commercial diagnostic kit (Asan Pharmaceutical, Hwasung, Republic of Korea) was used to quantify aspartate transaminase (AST) and alanine transaminase (ALT) levels in the plasma, following the instructions suggested by the manufacturers. Briefly, 5 μL of plasma was combined with 250 μL of either AST or ALT-specific solution, as supplied in the diagnostic kit. Following a 30 min incubation for AST or 60 min incubation of ALT at 37°C, the mixture was then blended with 250 μL of the respective coloring reagent (AST or ATL-specific, provided in the diagnostic kit). After a subsequent 20 min incubation at room temperature, 250 μL of 0.4 N NaOH was introduced to halt the reaction. Finally, the AST and ATL were quantified by measuring absorbance at 490 nm.
